# P-1804. Deep Mutational Scanning of the RSV Fusion Protein Reveals Mutational Constraint and Antibody Escape Mutations

**DOI:** 10.1093/ofid/ofaf695.1973

**Published:** 2026-01-11

**Authors:** Cassandra Simonich, Teagan E McMahon, Jesse Bloom

**Affiliations:** University of Washington/ Seattle Children's Hospital, Seattle, WA; Fred Hutch Cancer Center, Seattle, Washington; Fred Hutchinson Cancer Research Center, Seattle, Washington

## Abstract

**Background:**

Respiratory syncytial virus (RSV) is a leading cause of lower respiratory tract infections of infants and older adults. Recent advances in RSV prevention include new vaccines for adults including one to protect infants via passive transfer of maternal antibodies and a new monoclonal antibody (mAb) for infants that target the fusion protein (F). However, efforts to combat RSV must deal with antigenic variability in F which can lead to escape from prophylactic measures. Viral escape as a vulnerability of mAb prophylaxis has been demonstrated, and RSV surveillance efforts have identified circulating strains that contain mutations in key antigenic sites of F. Here we perform deep mutational scanning (DMS) of RSV F to comprehensively map the functional and antigenic effects of mutations.

**Figure:**
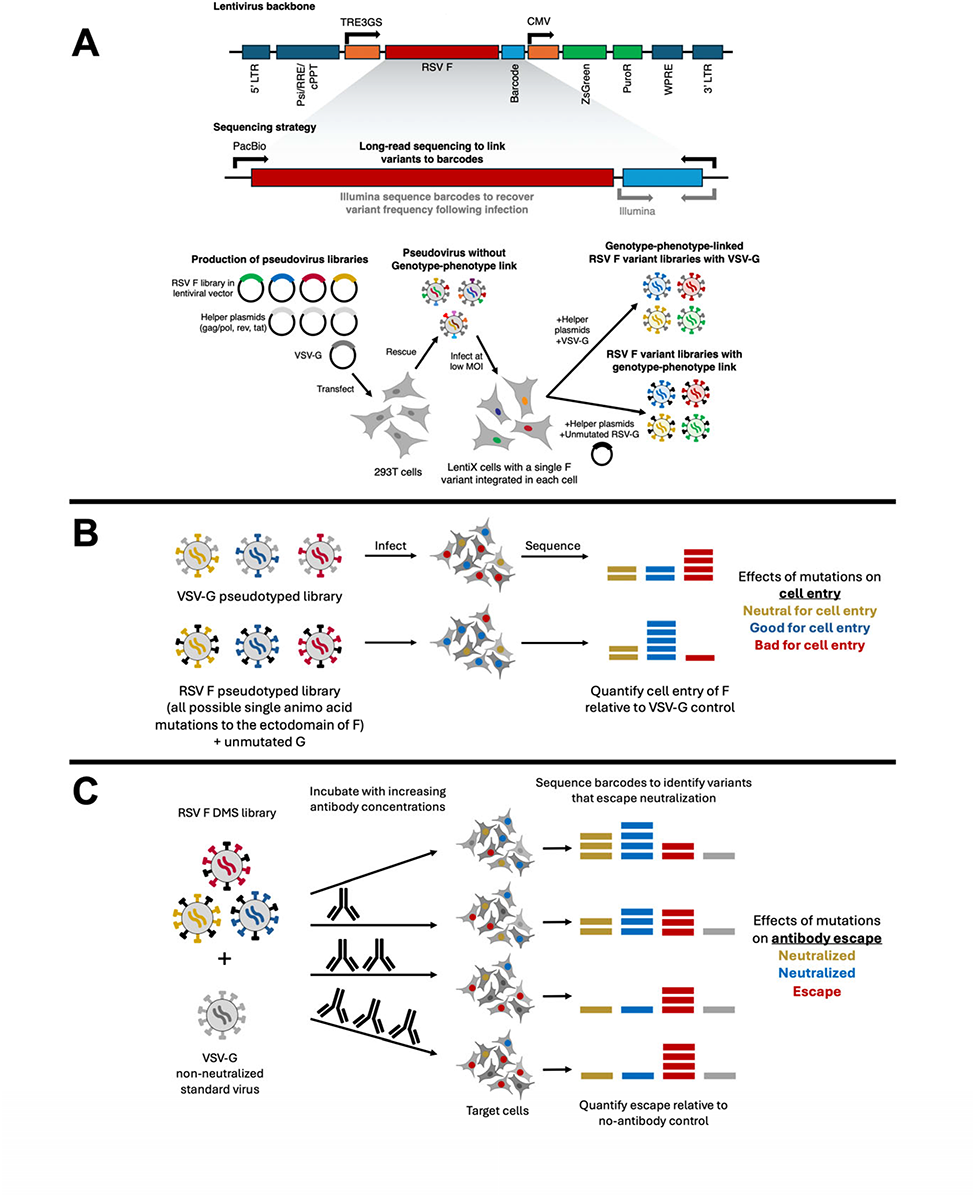
Graphical overview of RSV F deep mutational scanning for measuring cell entry and antibody escape (A) Strategy to make genotype-phenotype-linked pseudoviruses. A library of barcoded and mutagenized F variants in a lentiviral vector are transfected into 293T cells with additional lentiviral helper plasmids and a plasmid encoding VSV-G to create pseudoviruses that lack a genotype-phenotype link. To establish a link between the F on the virion surface and the genotype, we then infect LentiX cells at a low multiplicity of infection (MOI<0.01) to ensure a single integration per cell, and cells with integrated provirus are selected with puromycin creating a cell-stored library of F mutants in a lentiviral backbone. Transfection of helper plasmids into the cell-stored libraries plus either VSV-G or unmutated RSV G generates virions that have a single F protein variant on the surface and encode an identifying barcode in their genome. PacBio sequencing of the F gene and barcode from the library of singly-integrated cells is used to link variants in F with specific barcodes. For DMS experiments, Illumina sequencing of the short barcode region is used to identify the F variant. (B) Mapping mutational effects on F-mediated cell entry. Pseudoviruses with either RSV F + VSV-G (control condition) or RSV F + unmutated RSV G are used to infect target cells. Pseudoviruses with VSV-G on the surface infect cells regardless of which F variant is expressed on the surface of the virion. After infection, unintegrated viral DNA is extracted, and barcodes within each lentivirus vector are sequenced with Illumina. Reads from the RSV F + unmutated G infection condition are used to quantify cell entry relative to the VSV-G control. (C) Mapping effects of mutations on antibody neutralization. The pseudovirus library is incubated with increasing concentrations of antibody then used to infect target cells. Barcodes are recovered from infected cells and deep sequenced to quantify the ability of each variant to infect cells at each antibody concentration. A non-neutralized standard is used to convert the sequencing counts into the fraction of each variant neutralized at each antibody concentration. Mutants that increase in relative frequency in the presence of neutralizing antibodies compared to a no-antibody control are escape mutations.

**Figure:**
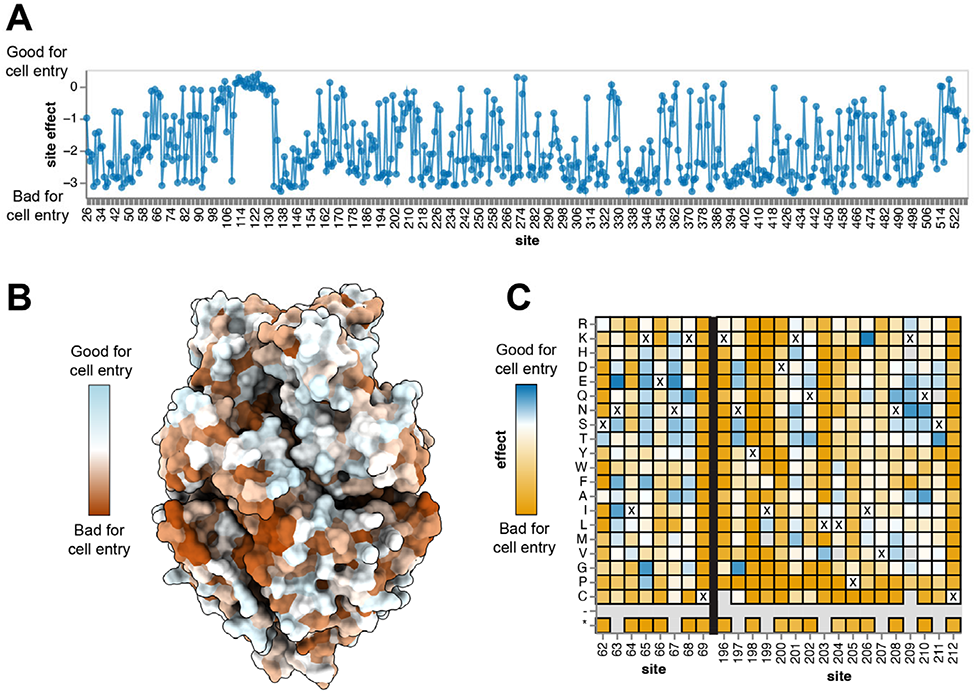
Effects of RSV F mutations on cell entry (A) Mean functional effect of all amino-acid mutations at each site in the RSV F ectodomain. Zero indicates no effect and entry comparable to the unmutated F, negative values indicate impaired entry, positive values indicate improved entry. (B) RSV F trimeric structure colored by the effect of mutations on cell entry (PDB 5c6b). Each site is colored by the mean effect of all amino-acid mutations at that site with orange indicating impaired cell entry, white indicating entry comparable to the unmutated F and blue indicating improved entry. (C) Cell entry effects of mutations in the nirsevimab binding site. For each mutation, the entry score reflects the cell entry efficiency of a pseudovirus with that F mutation relative to the unmutated F. Darker yellow indicates impaired entry, white indicates no effect and blue indicates improved entry. The wild-type amino acid at each site is indicated with a “X.” Mutations that were not measured are indicated with a light gray box.

**Methods:**

To investigate RSV antigenic evolution and map the evolutionary space accessible to RSV F, we utilize a high-throughput, pseudovirus platform to perform DMS of RSV F to measure how nearly all amino-acid mutations affect cell entry and antibody neutralization (Figure 1A-C). We made duplicate mutant libraries targeting all single amino-acid mutations of the F ectodomain.

**Figure:**
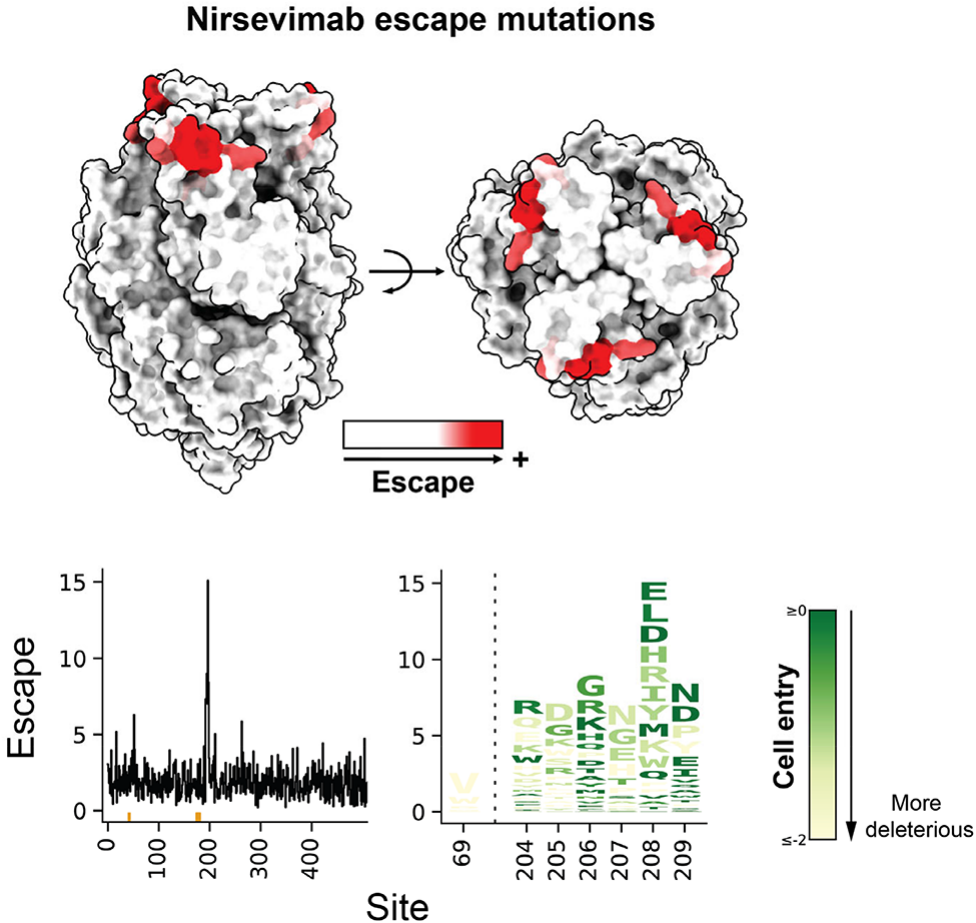
Nirsevimab escape mutations identified by DMS (A) Nirsevimab escape mutations mapped onto the structure of the RSV F trimer (PDB 5c6b). Sites where mutations cause strong escape are in darker red. (B) Key sites of escape for nirsevimab. The height of each letter in the logo plot is proportional to the escape caused by that amino-acid mutation, and letters are colored by the effect of that mutation on cell entry (dark green indicates well-tolerated, light yellow indicates impaired cell entry).

**Results:**

The two independent RSV F libraries each contain ∼9,500 amino-acid mutations and ∼47,000 unique barcoded F variants that each cover >99% of all possible mutations. Most F variants carried a single amino-acid mutation. Our DMS reveals regions of mutational constraint in RSV F likely due to protein folding and conformational change requirements for function (Figure 2A-B). Our study also identifies mutations at known antigenic regions that do not impact cellular entry, suggesting the possibility of viral escape from antibodies targeting these regions (Figure 2B-C). We map escape mutations for clinically relevant mAbs including nirsevimab (Figure 3). Nirsevimab escape mutations include mutations not previously identified and mutations that are functionally tolerated. These mutations exist among natural RSV sequences at low frequency.

**Conclusion:**

The DMS maps of mutational effects further our understanding of RSV F function and facilitate interpretation of the functional and antigenic consequences of F mutations observed by RSV surveillance efforts in the setting of more widespread use of vaccines and mAbs.

**Disclosures:**

Jesse Bloom, PhD, Apriori Bio: Advisor/Consultant|Apriori Bio: I am inventor on patents licensed to Apriori Bio by the Fred Hutch|GSK: Advisor/Consultant|Invivyd: Advisor/Consultant|Pfizer: Advisor/Consultant

